# Associations of coffee drinking with physical performance in the oldest-old community-dwelling men The Helsinki Businessmen Study (HBS)

**DOI:** 10.1007/s40520-020-01645-6

**Published:** 2020-07-08

**Authors:** Satu K. Jyväkorpi, Annele Urtamo, Mika Kivimäki, Timo E. Strandberg

**Affiliations:** 1 Department of Medicine, Clinicum and Helsinki University Hospital, University of Helsinki, Tukholmankatu 8 B, 00014 Helsinki, Finland; 2 Center for Life Course Health Research, University of Oulu, Oulu, Finland

**Keywords:** Coffee, Gait speed, Physical performance, Appendicular lean mass, Oldest-old men

## Abstract

**Background:**

Habitual coffee drinking has been associated with lower risk of various chronic diseases linked to poor physical performance.

**Objective:**

We explored cross-sectional associations between coffee consumption and physical performance among oldest-old community-dwelling men in the Helsinki Businessmen Study (HBS).

**Methods:**

A random sample of HBS survivors (*n* = 126, mean age 87 years) attended a clinic visit in 2017/2018, including measurements of body composition, physical performance [Short Physical Performance Battery (SPPB)], and cognition. Coffee consumption was retrieved from 3-day food diaries.

**Results:**

Coffee consumption was positively associated with higher gait speed (*p* = 0.003), SPPB score (*p* = 0.035), and chair rise points (*p* = 0.043). Association of coffee with gait speed remained after adjustment for age, waist circumference, physical activity, pulse rate, and high-sensitivity C-reactive protein.

**Conclusion:**

Higher coffee consumption was independently associated with better physical performance reflected as faster gait speed in oldest-old men.

## Background

Good physical function and performance are important for prevention of various health conditions and immobility among older adults [1]. In particular, low gait speed and chair stand have been associated with difficulties in performing activities of daily living (ADL) and poorer quality of life [2]. Knowledge of nutrition-related factors, physical function, and performance have been mainly limited to protein [3] and vitamin D intakes [4], specific foods such as fruit and vegetable and dairy products [5], and healthy dietary patterns such as the Mediterranean-type [6].

Coffee is one of the most widely consumed beverages in the world. It has an ergogenic potential as it is high in caffeine, which is a natural stimulant [7]. Habitual coffee consumption has been linked to reduced risk of type 2 diabetes [8], cardiovascular diseases [9], and Alzheimer’s disease [10], all of which are important contributors to impaired physical function and mobility [11].

A recent longitudinal study found that older coffee-drinking women had lower risk of immobility and ADL disability as compared to non-coffee drinkers [12]. Otherwise studies are scarce of physical performance, function, mobility, and coffee consumption in older people, especially men who on the average have better physical function than women of similar age.

## Aims

Our objective was to investigate associations of coffee consumption with physical performance of oldest-old home-dwelling men.

## Methods

In the Helsinki Businessmen Study (HBS), a socioeconomically homogenous cohort of men, born between 1919 and 1934, has been followed up since the 1960s [13]. In the present cross-sectional analysis, we report findings from the most recent clinic visit of a random sub-cohort of home-living survivors of HBS in 2017/2018. At the clinic visit, fasting venous samples were drawn, body mass index (BMI) was calculated (kg/m^2^), waist circumference measured, nutritional status assessed with the Mini Nutritional Assessment Short Form (MNA-SF) [14] and Short Physical Performance Battery (SPPB) test [15] performed as instructed, and gait speed (4-m walk, m/s) was measured. In addition, body composition analyzed using DXA-scans, and appendicular lean mass (ALM)/m^2^ calculated according to the classification by Gould et al. [16] and resting pulse was measured. Cognition was measured using The Montreal Cognitive Assessment (MOCA) Screening tool [17]. Laboratory analyses included high-sensitivity C-reactive protein (hs-CRP) to reflect inflammatory status. Daily coffee drinking, energy and macronutrient intakes, and protein distribution between daily meals were retrieved from 3-day food records. The participants were divided into three groups according to their daily coffee consumption (1) < 110 g, (2) 110–330 g, and (3) > 330 g, and variables classified to the coffee categories accordingly.

### Statistical analyses

We used descriptive statistics and analysis of covariance (ANCOVA) to investigate independent associations with coffee consumption. Adjustments were made for age, waist circumference, self-rated physical activity, pulse rate, and hs-CRP levels. Analyses were performed using the SPSS statistical program, version 24 (SPSS IBM, Armonk, NY, USA).

#### Selection of the covariates

Selection of covariates was based on previous research; waist circumference may indicate higher inflammatory load, metabolic problems, and poorer physical performance, inflammatory status measured by hs-CRP was selected as a covariate, because in previous research, high hs-CRP has been linked to lower physical performance in older people [18]. Pulse rate was selected as a covariate, because lower resting heart rate may indicate a higher cardiovascular fitness in the ANCOVA model.

## Results

Of the 130 men who attended the clinic, 126 participants (mean age 87 years) returned 3-day food records. Median daily coffee consumption was 220 g (IQ range 126–376). In the ANOVA test of linearity, consumption of coffee was not significantly associated with age, BMI, waist circumference, MNA-SF, physical activity, cognition, or hs-CRP. Linear association of coffee consumption was observed with gait speed (*p* = 0.003), SPPB (*p* = 0.035) and chair rise points (*p* = 0.043) (Table 1).

**Table 1:** Participant characteristics according to level of coffee consumption

Characteristics	Coffee groups			
	< 110 g of coffee per day (*n* = 15)	110–330 g of coffee per day (*n* = 74)	> 330 g of coffee per day (*n* = 37)	*p* value^a^
Age, years (SD)	88.3 (3.2)	87.3 (3.1)	87.2 (2.3)	0.327
BMI, kg/m^2^ (SD)	25.4 (2.3)	25.7 (2.8)	26.4 (2.7)	0.141
Waist circumference (SD]	103 (7.7)	101 (8.5)	102 (9.8)	0.997
MNA-SF (SD]	13 (1)	13 (1)	13 (1)	0.682
Cognition, MOCA test	22.4 (2.6)	23.6 (2.3)	23.2 (2.1)	0.584
Physically active, %	47	41	54	0.379
hs-CRP (SD)	3.1 (3.5)	2.7 (3.3)	2.1 (2.5)	0.252
SPPB (SD)	8.4 (3.4)	9.3 (2.3)	9.9 (2.1)	0.035
Gait speed, m/s (SD)	0.8 (0.2)	0.9 (0.2)	1.0 (0.2)	0.003
Chair rise, points (SD)	2.27 (1.5)	2.51 (1.3)	2.97 (1.1)	0.043
Grip strength, kg (SD)	28.7 (8.5)	31.5 (7.3)	30.2 (6.8)	0.856
Alm/m^2^ (SD)	6.97 (0.7)	7.04 (0.7)	7.30 (0.7)	0.144
Dietary intakes				
Energy, kcal [SD)	1456 [335)	1589 [315)	1639 [438)	0.123
Protein, g [SD)	67 (23)	75 (19)	73 (25)	0.593
g/kg BW/day [SD)	0.9 (0.3)	1.0 (0.3)	0.9 (0.3)	0.695
Vegetable protein, g [SD)	19 (10)	21 (6)	23 (7)	0.047
Animal protein, g [SD)	47 (18)	54 (18)	50 (23)	0.958
Protein distribution				
Breakfast, g (SD)	21 (12)	15 (7)	15 (6)	0.066
Morning snack, g (SD)	0	3 (5)	5 (8)	0.017
Lunch, g (SD)	21 (11)	23 (14)	23 (14)	0.597
Afternoon snack, g (SD)	3 (6)	5 (8)	5 (4)	0.547
Dinner, g (SD)	16 (18)	22 (18)	17 (17)	0.799
Evening snack, g (SD)	5 (7)	7 (7)	7 (6)	0.364
Carbohydrate, g (SD)	148 (36)	169 (42)	177 (47)	0.036
Starch, g (SD)	69 (19)	84 (21)	95 (32)	0.001
Sugar, g (SD)	21 (7)	26 (14)	23 (11)	0.918
Fiber, g (SD)	21 (7)	21 (8)	24 (10)	0.148
Fat, g (SD)	61 (26)	64 (20)	67 (21)	0.185
SFA, g (SD)	22 (7)	22 (7)	22 (9)	0.797
MUFA, g (SD)	23 (15)	24 (11)	25 (10)	0.490
PUFA, g (SD)	10 (6)	12 (4)	13 (6)	0.076

^a^The statistical significance of the hypotheses of linearity was evaluated for a trend using ANOVA and for non-evenly distributed variables with Kruskal—Wallis test, SD = standard deviation; BMI = body mass index; MNA-SF = Mini Nutritional Assessment Short Form; hs-CRP = high-sensitive C-reactive protein; IQR = Inter-quartile range; SPPB = Short Physical Performance Battery; m/s = meters per second; Alm = appendicular lean mass; m^2^ = meter square; d = day; kg = kilograms; The statistical significance of the hypotheses of linearity was evaluated for a trend using ANOVA; BW = Body Weight; SFA = Saturated fatty acids; MUFA = monounsaturated fatty acids; PUFA = polyunsaturated fatty acids^1^

Of nutritional variables, coffee drinking was linearly associated with higher plant protein (*p* = 0.047), carbohydrate (*p* = 0.036), and starch (*p* = 0.001) intakes (Table 2). In addition, those who drank more coffee, consumed more protein during morning snack (*p* = 0.017).

**Table 2:** ANCOVA models of factors associated with gait speed

Model	*B*	95% confidence interval		*p* value
		Lower bound	Upper bound	
Intercept	2.631	1.424	3.838	< 0.001
Coffee	0.000	0.000	0.000	0.013
Age	− 0.014	− 0.026	− 0.001	0.032
Waist circumference, cm	− 0.004	− 0.008	.001	0.088
Physical activity yes vs. no	0.096	.023	.169	0.011
Pulse	− 0.004	− 0.008	− 0.001	0.013
hs-CRP	− 0.016	− 0.028	− 0.005	0.005
Adjusted *R*^2^	**0.244**			

Bold value indicates the amount of observed variation that can be explained by the model’s inputs

*BMI* body mass index, *Hs-CRP* high-sensitivity C-reactive protein, *R*^*2*^ *R* squared

Coffee consumption was associated with faster gait speed in ANCOVA model controlling for age, physical activity, pulse rate, hs-CRP, and waist circumference (Table 2).

## Discussion

In the oldest-old community-dwelling men, higher consumption of coffee was associated with better physical performance and faster gait speed. This linear association remained statistically significant after adjusting for multiple covariates, a finding which is consistent with the hypothesis that moderate coffee drinking may be beneficial for physical performance in oldest-old men.

Coffee is naturally high in caffeine, which has an ergogenic potential. In fact, high amount of caffeine is known to enhance physical performance in athletes [7]. Although habitual coffee drinking can enhance performance, the daily amount of coffee consumption in our older participants was relatively low [median] about two small cups or 220 g/day. As some studies have reported that athletes did not get extra benefit for their performance by consuming much higher amounts of coffee per day [19], a recent study showed that even low acute dose of caffeine (2 mg/kg for 80 kg individual) that equal to approximately 200 g of coffee a day may produce substantial improvements in lower body muscle endurance [20]. Coffee also contains several bioactive compounds such as polyphenols and minerals and up to 1000 other bioactive components that could potentially affect performance and prevent chronic diseases. In observational studies, moderate coffee consumption has been associated with lower incidence of type 2 diabetes, and cardiovascular and Alzheimer’s diseases, all known contributors to decline in physical function of older people [12]. Furthermore, a recent longitudinal study of older women found that coffee consumption of ≥ 2 cups a day as compared to zero consumption was associated with reduced immobility [13]. The researchers also found that women with type 2 diabetes who consumed ≥ 2 cups coffee a day had lower risk of ADL disability [13]. Our results in men support those findings as both gait speed and SPPB-test scores were higher in those who consumed more coffee. It is currently unknown whether contents of caffeine, polyphenol, mineral, other bioactive ingredients, or a combination of all of them contribute to the potential health effects of coffee consumption.

Among dietary factors, carbohydrate, starch, and plant protein intakes were higher among those who drank more coffee. Consequently, the participants drinking more coffee also consumed more cereal-based food such as bread, porridge, or salty pastries as compared to those who drank less. Coffee drinking was also associated with more snacking in our study, especially snacking between breakfast and lunch was more frequent among those who drank more coffee. In line with these findings, higher coffee consumption tended to be associated with higher energy intake, higher BMI, and greater appendicular lean mass. Then, a question arises whether higher coffee consumption leads to better performance via increased energy content of the diets and better nutrition in these oldest-old men.

## Strengths and limitations

The strengths of our study are the robust findings of coffee consumption and physical performance, although our study sample was relatively small. Obviously, the surviving participants of HBS differ in many ways from the general older population due to their high socioeconomic status. There is interesting evidence that sex would modify the association between coffee consumption and cognition, with stronger effect among females [21]. This could also apply to physical performance, but unfortunately potential differences between sexes could not be compared in our study. The cross-sectional design of the study prevents drawing conclusions about causal relationships. It also is possible that the observed associations are attributable to bias and confounding by unmeasured or imprecisely measured risk factors. Finally, hypersensitivity or intolerance to coffee may be linked to health problems, contributing to a spurious association between coffee consumption and physical performance.

## Conclusion

We observed an independent association between higher consumption of coffee and better physical performance and faster gait speed, which are important determinants of health and survival of older people. As coffee intakes are modifiable, these findings encourage further intervention studies to determine whether moderate coffee drinking could be recommended as part of a healthy diet for the oldest-old.

## References

[1] Mijnarends DM, Luiking YC, Halfens R, Evers S, Lenaerts E, Verlaan S, Wallace M, Schols J, Meijers J (2018). Muscle, health and costs: a glance at their relationship. J Nutr Health Aging.

[2] van Lummel RC, Walgaard S, Pijnappels M, Elders PJM, Garcia-Aymerich J, van Dieen JH, Beek PJ (2015). Physical performance and physical activity in older adults: associated but separate domains of physical function in old age. PLoS ONE.

[3] Tieland M, van de Rest O, Dirks ML, van der Zwaluw N, Mensink M, van Loon LJ, de Groot LC (2012). Protein supplementation improves physical performance in frail elderly people: a randomized, double-blind, placebo-controlled trial. J Am Med Dir Assoc.

[4] Dewansingh P, Melse-Boonstra A, Krijnen WP, van der Schans CP, Jager-Wittenaar H, van den Heuvel EGHM (2018). Supplemental protein from dairy products increases body weight and vitamin D improves physical performance in older adults: a systematic review and meta-analysis. Nutr Res.

[5] Houston DK, Stevens J, Cai J, Haines PS (2005). Dairy, fruit, and vegetable intakes and functional limitations and disability in a biracial cohort: the atherosclerosis risk in communities study. Am J Clin Nutr.

[6] Struijk EA, Guallar-Castillon P, Rodriguez-Artalejo F, Lopez Garcia E (2016). Mediterranean dietary patterns and impaired physical function in older adults. J Gerontol A Biol Sci Med Sci.

[7] Higgins S, Straight CR, Lewis RD (2016). The Effects of preexercise caffeinated coffee ingestion on endurance performance: an evidence-based review. Int J Sport Nutr Exerc Metab.

[8] Ding M, Bhupathiraju SN, Chen M, van Dam RM, Hu FB (2014). Caffeinated and decaffeinated coffee consumption and risk of type 2 diabetes: a systematic review and a dose-response meta-analysis. Diabetes Care.

[9] Ding M, Bhupathiraju SN, Satija A, van Dam RM, Hu FB (2014). Long-term coffee consumption and risk of cardiovascular disease: a systematic review and a dose-response meta-analysis of prospective cohort studies. Circulation.

[10] Eskelinen MH, Ngandu T, Tuomilehto J, Soininen H, Kivipelto M (2009). Midlife coffee and tea drinking and the risk of late-life dementia: a population-based CAIDE study. J Alzheimers Dis.

[11] Stuck AE, Walthert JM, Nikolaus T, Büla CJ, Hohmann C, Beck JC (1999). Risk factors for functional status decline in community living elderly people: a systematic literature review. Soc Sci Med.

[12] Machado-Fragua MD, Struijk EA, Graciani A, Guallar-Castillon P, Rodríguez-Artalejo F, Lopez-Garcia E (2019). Coffee consumption and risk of physical function impairment, frailty and disability in older adults. Eur J Nutr.

[13] Strandberg TE, Salomaa V, Strandberg AY, Vanhanen H, Sarna S, Pitkälä K, Rantanen K, Savela S, Pienimäki T, Huohvanainen E, Stenholm S, Räikkönen K, Tilvis RS, Tienari PJ, Huttunen J (2016). Cohort profile: The Helsinki Businessmen Study (HBS). Int J Epidemiol.

[14] Vellas B, Guigoz Y, Garry PJ, Nourhashemi D, Bennahum D, Launque S, Albarede L (1999). The Mini Nutritional Assessment [MNA] and its use in grading the nutritional state of elderly patients. Nutrition.

[15] Guralnik JM, Simonsick EM, Ferrucci L, Glynn RJ, Berkman LF, Blazer DG, Scherr PA, Wallace RB (1994). A short physical performance battery assessing lower extremity function: association with self-reported disability and prediction of mortality and nursing home admission. J Gerontol.

[16] Gould H, Brennan SL, Kotowicz MA, Nicholson GC, Pasco JA (2014). Total and appendicular lean mass reference ranges for Australian men and women: the Geelong osteoporosis study. Calcif Tissue Int.

[17] Julayanont P, Tangwongchai S, Hemrungrojn S, Tunvirachaisakul C, Phanthumchinda K, Hongsawat J, Suwichanarakul P, Thanasirorat S, Nasreddine ZS (2015). The Montreal cognitive assessment-basic: a screening tool for mild cognitive impairment in illiterate and low-educated elderly adults. J Am Geriatr Soc.

[18] Cesari M, Penninx BWJH, Pahor M, Lauretani F, Corsi AM, Williams GR, Guralnik JM, Ferrucci L (2004). Inflammatory markers and physical performance in older persons: the in CHIANTI study. J Gerontol.

[19] Tunnicliffe JM, Erdman KA, Reimer RA, Lun V, Shearer J (2008). Consumption of dietary caffeine and coffee in physically active populations: physiological interactions. Nutr Metab.

[20] Grgic J, Sabol F, Venier S, Mikulic I, Bratkovic N, Schoenfeld BJ, Pickering C, Bishop DJ, Pedisic Z, Miculic P (2019). What dose of caffeine to use: acute effects of 3 doses of caffeine on muscle endurance and strength. Int J Sports Physiol Perform.

[21] Richie K, Carriere A, de Mendonca F, Porter JF, Dartigues O, Rouaud P, Barberger-Gateau M, Ancelin L (2007). The neuroprotective effects of caffeine—a prospective population study (the Three City Study). Neurology.

